# The effect of gender norms on the association between violence and hope among girls in the Democratic Republic of the Congo

**DOI:** 10.1017/gmh.2016.31

**Published:** 2017-01-09

**Authors:** L. Stark, K. Asghar, S. Meyer, G. Yu, T. Bakemore, C. Poulton, K. Falb

**Affiliations:** 1 Department of Population and Family Health, Columbia University Mailman School of Public Health, 60 Haven Ave B-4 Suite 432, New York, NY, USA; 2 New York University College of Nursing, 433 First Avenue, New York, NY, USA; 3 International Rescue Committee, 35/01 Ave P-E Lumumba, Bukavu, DRC; 4 International Rescue Committee, Place de la Vieille aux Bles 16, Bruxelles, Belgium; 5 International Rescue Committee, 122 E 42nd St, New York, NY, USA

**Keywords:** Attitudes toward intimate partner violence, etiology, gender norms, hope, resilience, violence

## Abstract

**Background.:**

Girls at early stages of adolescence are vulnerable to violence victimization in humanitarian contexts, but few studies examine factors that affect girls’ hope in these settings. We assessed attitudes toward traditional gender norms as an effect modifier of the relationship between violence exposure and future orientation in displaced girls.

**Methods.:**

Secondary analysis, using multivariable regression of cross-sectional data from girls ages 10–14 in South Kivu, Democratic Republic of the Congo. Key variables of interest were attitudes toward intimate partner violence (IPV), Children's Hope Scale (CHS) score, and exposure to physical, emotional, and sexual violence within the last 12 months. Additional covariates included age, educational status, and territory.

**Results.:**

The interaction of exposure to violence and attitudes toward IPV magnified the association between violence exposure and lower CHS score for physical violence (*β* = −0.09, *p* = 0.040) and unwanted sexual touching (*β* = −0.20, *p* = 0.003) among girls age 10–14, when adjusting for other covariates. The interaction of exposure to violence and attitudes toward IPV magnified the association between violence exposure and lower CHS score for forced sex (*β* = −0.22, *p* = 0.016) among girls age 13–14, when adjusting for covariates. Findings for emotional violence, any form of sexual violence, and coerced sex trended toward lower CHS scores for girls who reported higher acceptance of IPV, but did not reach significance.

**Conclusions.:**

Findings support the utility of gender norms-transformative programming in increasing resilience of girls who have experienced sexual violence in humanitarian contexts.

## Introduction

Exposure to interpersonal violence – the intentional use of physical force or power against another, which may result in injury, death or psychological harm – increases the likelihood that individuals will experience symptoms of depression, post-traumatic stress (PTS), and anxiety (Jaycox *et al*. 2002; van der Kolk, 2014). These associations are higher in women than men (Tolin & Foa, 2006). There is also specific evidence demonstrating that violence victimization is a predictor of poor mental health outcomes in adolescence (Mollica *et al*. 1997; Turner *et al*. 2006). Girl survivors, again, are at higher risk for symptoms of depression and PTS than boy survivors (Jaycox *et al*. 2002).

Poor mental health, particularly depression, has also been linked to low levels of hope among both adults and children (Kazdin *et al*. 1983; Thimm *et al*. 2013). ‘Hope’ is a construct that includes optimism about the future and belief in being able to reach one's goals (Snyder *et al*. 1997). Hope can be an indicator of adolescent well-being and resilience (Gilman *et al*. 2006), and greater levels of hope have been linked to improved health and educational attainment (Gushue *et al*. 2006). Studies have indicated that hope has, in many cases, a stronger association with suicide attempts and overall well-being than does depression (Kazdin *et al*. 1983; Savahl *et al*. 2013).

To date, hope has most often been examined as a moderator or mediator, such as in the relationship between trauma and mental health (Zhang *et al*. 2009; Geiger & Kwon, 2010), rather than as an outcome in itself. The limited research that has examined hope as an outcome has examined factors such as self-esteem, depression, social-support, and stress as predictors of hope in adolescence (Yarcheski & Mahon, 2016). While there is an established evidence base linking violence exposure to mental health sequelae, the relationship between violence exposure and hope has yet to be explored in depth. Given that hope may be a strong determinant of well-being (Savahl *et al*. 2013), and has also been identified as a potentially important protective and promotive factor specifically for children in humanitarian settings (for example, in Eggerman & Panter-Brick, 2010), understanding predictors of hope may contribute to efforts to improve health among survivors of violence.

Of further interest is the role that gender norms may play in moderating the relationship between violence exposure and one's hope about the future. There is an established knowledge base demonstrating that acceptance of more traditional gender roles is associated with lower well-being among adults (van de Vijver, 2007), and identification with a traditionally feminine gender role has been linked to higher depression amongst adolescents (Broderick & Korteland, 2002). There is also evidence linking attitudinal acceptance of intimate partner violence (IPV) with increased violence exposure in adolescents (Lichter & McCloskey, 2004).

This article is, to our knowledge, a first attempt to examine the moderating effect of attitudes toward IPV on the relationship between violence exposure and hope amongst displaced girls living in South Kivu, Democratic Republic of the Congo (DRC).

Role congruity theory (Eagly & Diekman, 2005) asserts that people are ‘rewarded’ for behaving in accordance with the social role ascribed to their gender, and ‘punished’ for behavior that violates those social roles (Diekman & Goodfriend, 2006). According to this framework, asking individuals about their attitudes toward IPV specifically gauges prescriptive norms on the acceptability of violence (Lichter & McCloskey, 2004; Diekman & Goodfriend, 2006; Eagly & Koenig, 2008). Changing community-level gender norms is a large component of global violence prevention programming, but the relationship between these prescriptive norms and hope for one's future – especially among those who may have experienced violence victimization – is poorly understood.

Having a better understanding of the relationships between interpersonal violence exposure, prescriptive norms related to acceptability of violence, and future orientation has implications for populations affected by widespread exposure to violence as well as for practitioners seeking to promote well-being for survivors. In this article, we hypothesize that attitudes toward IPV moderate the relationship between violence exposure and hope for one's future.

## Methods

### Setting

Eastern DRC has been affected by conflict for nearly 20 years. Together, North, and South Kivu are currently home to over 68% of the displaced population of the DRC (UNOCHA, 2008). The proliferation of armed groups using sexual violence as a targeted weapon, separation of families, the collapse of traditional community protection mechanisms, and displacement have all contributed to increased risk for adolescent girls (Bell, 2006; Stark *et al*. 2016). According to the Demographic and Health Survey (2007), 24% of girls aged 15–19 years had already begun child-bearing. Such high adolescent fertility rates have important negative consequences for the health and well-being of adolescent girls (Hindin & Fatusi, 2009). Additionally, girls’ exclusion from decision-making processes and lack of control over their own lives also limit their physical, emotional, and social development.

### Participants

Participants were from a baseline evaluation of the Creating Opportunities through Mentorship, Parental Involvement, and Safe Spaces (COMPASS) program, conducted in 14 villages across the Kabare, Uvira, and Walungu districts in South Kivu, DRC. Girls age 10–14 who spoke French, Swahili, or Mashi, were invited to participate in the program, and completed a baseline survey prior to starting the program. In total, 929 girls were assessed for eligibility, 886 were found to be eligible, and 869 girls completed the survey (98% completion rate). Recruitment and enrollment are described in detail elsewhere (see Falb *et al*. 2016).

### Procedure

Girls who had registered for the life skills program were invited to complete interviews at established safe spaces in each of the 14 villages. Consent was obtained from caregivers, and assent from girls, prior to completing the survey. A confidential survey, taking approximately 1 h, was administered by data collectors matched to participants by gender and language. Less sensitive questions were administered using Computer-Assisted Personal Interview. Adolescents age 13–14 (*N* = 377) answered more sensitive questions on violence, relationship status, and sexual health using Audio Computer-Assisted Self-Interview programming. Respondents were provided information about available referral services after the interview. All components of the research were reviewed and approved by Columbia University's Human Subjects Protection committee, and by the Ministry of Gender in the DRC.

### Measures

Demographic data were collected on age, educational status and relationship status. Age was measured as a continuous variable. Years of education completed was measured as a continuous variable, with girls who reported never attending school categorized as completing zero years of school. Only girls ages 13–14 were asked about their relationship status, based on piloting feedback that these questions were deemed sensitive for younger girls as they infer sexual activity. Respondents age 13–14 who reported living with their husband or a ‘man as if married’ were categorized as living with an intimate partner.

Binary questions on violence were adapted from ICAST and VACS questionnaires (Reza *et al*. 2009; Zolotor *et al*. 2009). Physical violence was defined as being hit or beaten in the previous 12 months. Emotional violence was operationalized as being screamed at loudly or aggressively in the previous 12 months. Sexual violence was operationalized as experiencing unwilling (forced) sex, unwanted sexual touching, or verbal coercion (using influence or authority to threaten or pressure respondent to have sex) in the previous 12 months. As above, only girls age 13–14 were asked about forced sex.

Prescriptive norms related to attitudes about IPV were assessed through a composite variable summing response to five items from UNICEF's MICS (Multiple Indicator Cluster Survey) module. The module asks about acceptability of husbands beating wives for failing to adhere to traditional gender norms, not caring for her children in the proper way, and refusing to have sex, for example. The composite variable has a range of 0–5, where 0 indicates disagreement with all five statements on acceptability of IPV and 5 indicates agreement with all five statements.

Finally, hope for the future was assessed by the six-item Children's Hope Scale (CHS: Snyder *et al*. 1997). The CHS was developed to measure hope, conceptualized as the belief in one's ability to initiate and work toward goals, and is meant to capture the underlying components of a ‘pathway’ – perceived ability to produce a feasible route to the goal, and ‘agency’ or motivation to follow said route (Snyder *et al*. 1997). The instrument uses a five-point Likert scale, and scores are determined by averaging the six items for each participant.

The CHS was originally normed in different adolescent populations (ages 9–14) in the USA, and showed good internal consistency (Cronbach's *α* = 0.77) and test–retest reliability after 1 month (*r* = 0.71, *p* < 0.001) (Snyder *et al*. 1997). A subsequent validation study in a US population also showed high internal consistency (*α* = 0.83–0.84) (Valle *et al*. 2004). The scale shows fair convergent validity with measures of perceived competence and control, self-worth and self-esteem, and resilience (Snyder *et al*. 1997; Jovanović, 2013).

Several studies had previously employed the CHS to measure psychosocial wellbeing in humanitarian settings. These include conflict-afflicted regions of Burundi, Indonesia, Israel, Lebanon, and Nepal (Kasler *et al*. 2008; Tol *et al*. 2008, 2014; Jordans *et al*. 2010; Morley & Kohrt, 2013; Ayyash *et al*. 2016). Psychometric properties of the scale were demonstrated in several of these populations, with fairly high internal consistency (*α* range 0.622–0.71) and test–retest reliability (*r* range 0.667–0.95) (Tol *et al*. 2008, 2014; Jordans *et al*. 2010; Ayyash *et al*. 2016).

In the present study, cognitive interviewing techniques were used to test the scales in a pilot sample prior to baseline data collection (Falb *et al*. 2016).

### Statistical analyses

Linear regression was used to assess independent relationships between: (1) forms of violence and CHS score; (2) attitudes toward IPV and CHS score; and (3) forms of violence and attitudes toward IPV. Linear regression was also used to examine the moderating effect of attitudes toward IPV on the association between exposure to violence and hope score after adjusting for age, education level, and district. Living with an intimate partner was added to the model examining forced sex. Adjusted analyses included multivariable linear regression. Since the primary focus of this secondary analysis was to determine direction rather than magnitude of the hypothesized relationships, the regressions did not use standardized betas. All analyses were completed using SAS Version 9.4 (SAS Institute, Cary, NC).

## Results

The average age of study participants was 12.03 years (s.d. 1.50), and 80% of girls (*N* = 691) had ever attended school. Of the 377 girls ages 13–14 who were asked about their relationship status, 16.44% reported living with their husband or living with a man as if married. Almost 70% of girls reported being unmarried and 6.63% of girls were married but not living with their partner (for further demographic information on study participants, see Stark *et al*. 2016, forthcoming).

In terms of attitudes and experiences of violence, nearly 15% of girls (*N* = 127) reported that IPV was acceptable in all examples provided. Approximately 40% of participants reported being hit or beaten in the last 12 months (*N* = 340), and 39.86% reported emotional violence (*N* = 346). Twenty-three per cent of the sample reported experiencing sexual violence (*N* = 200), which included 13.58% of girls who reported experiencing unwanted sexual touching (*N* = 118); 12.77% who reported having been coerced into sex (*N* = 111); and (among girls age 13–14) 15.38% who reported having experienced forced sex (*N* = 58).

Average score on the CHS was 2.34 (range 1–5), demonstrating a low overall self-perception of girls’ ability to achieve goals. The alpha for this scale was 0.84, indicating high reliability in this context.

Adolescent girls who had been exposed to violence in the previous 12 months had significantly lower hope scores than those who had not been exposed to violence (*p* < 0.01 for all associations, see Table 1). Violence exposure was also significantly associated with more accepting attitudes toward IPV. Additionally, adolescents who held more accepting attitudes toward IPV were significantly more likely to report lower levels of hope (*β* = −0.09, *p* < 0.001). As such, attitudes toward IPV met the criteria for effect modification.

**Table 1.** Estimates of independent relationships between violence exposure, hope, and attitudes toward intimate partner violence (IPV)

	Children's hope scale score				Accepting attitudes toward IPV			
	*β*	s.e.	95% CI	*p*	*β*	s.e.	95% CI	*p*
Attitudes toward IPV	−0.09	0.02	(−0.13 to −0.05)	<0.001				
Exposure to violence								
Physical violence	−0.43	0.08	(−0.58 to −0.27)	<0.001	0.97	0.12	(0.74–1.20)	<0.001
Emotional violence	−0.39	0.08	(−0.54 to −0.24)	<0.001	0.77	0.12	(0.54–1.01)	<0.001
Any form of sexual violence	−0.32	0.09	(−0.50 to −0.15)	<0.001	0.91	0.14	(0.64–1.18)	<0.001
Unwanted sexual touching	−0.41	0.11	(−0.62 to −0.20)	<0.001	1.12	0.17	(0.79–1.45)	<0.001
Coerced sex	−0.32	0.11	(−0.53 to −0.11)	0.003	1.10	0.17	(0.76–1.44)	<0.001
Forced sex^a^	−0.48	0.16	(−0.79 to −0.17)	0.003	0.43	0.23	(−0.03 to 0.89)	0.064

a Girls age 13–14 only.

Each form of violence (physical violence, emotional violence, and any form of sexual violence, including unwanted sexual touching, coerced sex, and forced sex) was placed into a model with attitudes toward IPV (AIPV) and an interaction term for violence and IPV attitudes:

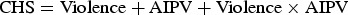


Results of this unadjusted model are in Table 2. The interaction between violence exposure and attitudes toward IPV modified the relationship between violence exposure and hope for respondents who experienced any form of sexual violence (*p* = 0.021), including unwanted sexual touching (*p* = 0.002) and forced sex (*p* = 0.015) in the previous 12 months. The interaction was associated with marginally lower scores on the CHS for physical violence (*p* = 0.051) and emotional violence (*p* = 0.055). While the interaction was associated with lower scores on the CHS for verbally coerced sex, this association did not achieve significance.

**Table 2.** Effect of violence exposure and attitudes toward intimate partner violence (IPV) on hope^a^

Predictor	*β*	s.e.	*p*	95% CI
Physical violence				
Intercept	2.54	0.08	<0.001	(2.39–2.69)
Physical violence	−0.13	0.15	0.382	(−0.4253 to 0.16)
Attitudes toward IPV	−0.02	0.03	0.536	(−0.08 to 0.04)
Physical violence × attitudes	−0.09	0.05	0.051	(−0.18 to 0.001)
Emotional violence				
Intercept	2.55	0.08	<0.001	(2.39 to 2.70)
Emotional violence	−0.11	0.14	0.438	(−0.39 to 0.17)
Attitudes toward IPV	−0.03	0.03	0.320	(−0.09 to 0.03)
Emotional violence × attitudes	−0.09	0.05	0.055	(−0.18 to 0.002)
Sexual violence in last 12 months				
Intercept	2.50	0.07	<0.001	(2.36–2.65)
Any form of sexual violence	0.12	0.18	0.528	(−0.24 to 0.47)
Attitudes toward IPV	−0.05	0.03	0.078	(−0.10 to 0.01)
Sexual violence × attitudes	−0.13	0.05	0.021	(−0.23 to −0.02)
Unwanted sexual touching				
Intercept	2.47	0.07	<0.001	(2.34–2.61)
Unwanted sexual touching	0.38	0.26	0.13	(−0.11 to 0.88)
Attitudes toward IPV	−0.04	0.02	0.06	(−0.09 to 0.002)
Unwanted sexual touching × attitudes	−0.22	0.07	0.002	(−0.36 to −0.08)
Coerced sex				
Intercept	2.50	0.07	<0.001	(2.36–2.63)
Coerced sex	0.10	0.25	0.678	(−0.39 to 0.60)
Attitudes toward IPV	−0.06	0.02	0.016	(−0.11 to −0.01)
Coerced sex × attitudes	−0.10	0.07	0.132	(−0.24 to 0.03)
Forced Sex				
Intercept	2.38	0.12	<0.001	(2.14–2.62)
Forced sex	0.32	0.32	0.512	(−0.42 to 0.84)
Attitudes toward IPV	0.04	0.04	0.807	(−0.07 to 0.09)
Forced sex × attitudes	−0.23	0.09	0.015	(−0.42 to −0.04)

a Unadjusted model, examining interaction of attitudes toward IPV with the six different forms of violence measured.

Unadjusted models that had demonstrated significant associations were further examined through adjusted analysis. Since the interaction terms for attitudes toward IPV and physical and emotional violence exposure were marginally significant, these two forms of violence exposure were also examined in the adjusted analysis. Because lower age and fewer years of education completed were independently associated with most violence exposures, and score on the CHS (age *p* = 0.330, school *p* ≦ 0.001), these factors were added into the model. District was also added into the model to adjust for other potential social norms and community-level factors that may influence girls’ attitudes toward IPV, with Walungu as the reference group. The adjusted model is as follows:

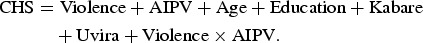


Additionally, a modified model was developed to examine the relationship between forced sex, attitudes toward IPV, and hope for girls age 13–14. Because intimate partners are the primary perpetrators of violence in this sample (Stark *et al*., forthcoming), and questions about living with an intimate partner were only asked to girls age 13–14, the original adjusted model was adapted to adjust for living with an intimate partner, and is as follows:

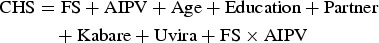


Residual graphs for the interaction model (Table 3) demonstrated random distribution of residuals, and non-violation of the homoscedasticity assumption. A one-point increase in attitudes accepting of IPV was associated with a 0.09-point reduction in hope. Thus, the acceptance of IPV magnified and strengthened the negative association of the CHS score difference between non-exposure (*β* of physical violence = −0.09) and exposure to physical violence (*β* of physical violence + *β* of interaction = −0.18), after controlling for age, years of school completed, and district. In the adjusted model, the interaction between exposure to emotional violence and attitudes toward IPV was not a significant moderator of CHS score.

**Table 3.** Effect of exposure to sexual violence and attitudes toward intimate partner violence (IPV) on hope, adjusted^a^^,^^b^

Predictor	*β*	s.e.	*p*	95% CI
Physical violence in last 12 months				
Intercept	3.07	0.32	<0.0001	(2.43–3.70)
Physical violence	−0.09	0.14	0.548	(−0.36 to 0.19)
Attitudes toward IPV	−0.02	0.03	0.605	(−0.07 to 0.04)
Age	−0.05	0.03	0.075	(−0.10 to 0.005)
Years of school completed	0.09	0.02	<0.0001	(0.05–0.12)
Kabare (district variable)	−0.30	0.15	0.038	(−0.59 to −0.02)
Uvira (district variable)	−0.08	0.15	0.611	(−0.37 to 0.22)
Physical violence × attitudes	−0.09	0.05	0.040	(−0.18 to 0.004)
*R*^2^	0.13			
Emotional violence in last 12 months				
Intercept	3.04	0.33	<0.0001	(2.39–3.68)
Emotional violence	−0.17	0.14	0.221	(−0.44 to 0.10)
Attitudes toward IPV	−0.04	0.03	0.210	(−0.09 to 0.02)
Age	−0.04	0.03	0.121	(−0.09 to 0.01)
Years of school completed	0.08	0.02	<0.0001	(0.04–0.12)
Kabare (district variable)	−0.31	0.15	0.034	(−0.60 to −0.02)
Uvira (district variable)	−0.04	0.15	0.797	(−0.33 to 0.25)
Emotional violence × attitudes	−0.06	0.04	0.148	(−0.15 to 0.02)
*R*^2^	0.13			
Sexual violence in last 12 months				
Intercept	2.78	0.34	<0.0001	(2.11–3.45)
Sexual violence	0.02	0.18	0.924	(−0.33 to 0.37)
Attitudes toward IPV	−0.05	0.03	0.045	(−0.10 to 0.001)
Age	−0.03	0.03	0.244	(−0.09 to 0.02)
Years of school completed	0.08	0.02	<0.0001	(0.04–0.12)
Kabare (district variable)	−0.22	0.16	0.166	(−0.53 to 0.09)
Uvira (district variable)	0.05	0.16	0.739	(−0.26 to 0.37)
Sexual violence × attitudes	−0.09	0.05	0.088	(−0.20 to 0.01)
*R*^2^	0.11			
Unwanted sexual touching				
Intercept	2.90	0.34	<0.0001	(2.24–3.57)
Unwanted sexual touching	0.31	0.25	0.205	(−0.17 to 0.80)
Attitudes toward IPV	−0.04	0.02	0.069	(−0.09 to 0.003)
Age	−0.04	0.03	0.119	(−0.10 to 0.01)
Years of school completed	0.09	0.02	<0.0001	(0.05–0.13)
Kabare (district variable)	−0.26	0.15	0.092	(−0.56 to 0.04)
Uvira (district variable)	0.01	0.16	0.959	(−0.30 to 0.31)
Unwanted sexual touching × attitudes	−0.20	0.07	0.003	(−0.34 to −0.07)
*R*^2^	0.13			
Forced sex^c^				
Intercept	1.24	1.74	0.478	(−2.19 to 4.67)
Forced sex	0.28	0.32	0.390	(−0.36 to 0.91)
Attitudes toward IPV	0.02	0.04	0.694	(−0.06 to 0.10)
Age	0.07	0.13	0.587	(−0.18 to 0.32)
Years of school completed	0.09	0.03	0.001	(0.03–0.14)
Kabare (district variable)	−0.18	0.29	0.526	(−0.75 to 0.39)
Uvira (district variable)	0.04	0.30	0.901	(−0.55 to 0.62)
Living with partner	−0.08	0.17	0.654	(−0.41 to 0.26)
Forced sex × attitudes	−0.22	0.09	0.016	(−0.41 to −0.04)
*R*^2^	0.14			

a Adjusted for age, education status, and district.

b Since the interaction term did not significantly modify the relationship between coerced sex and hope in the unadjusted model, these forms of violence were excluded from analysis in the adjusted model.

c Girls age 13–14 only. Adjusted for living with intimate partner in addition to age and education status.

Similarly, a one-point increase in attitudes accepting of IPV was associated with a 0.20-point reduction in hope. This again magnified and strengthened the negative association of the CHS score difference between non-exposure (*β* = 0.31) and exposure to unwanted sexual touching (*β* of unwanted touching + *β* of interaction = 0.07), after controlling for age, years of school completed, and district. In the adjusted model, the interaction between exposure to any form of sexual violence and attitudes toward IPV was not a significant moderator of CHS score.

Among girls age 13–14, a one-point increase in attitudes accepting of IPV was associated with a 0.22-point reduction, thus magnifying and strengthening the negative association of the CHS score difference between non-exposure (*β* = 0.28) and exposure to forced sex (*β* of forced sex + *β* of interaction = 0.06), after controlling for age, years of school completed, district, and living with an intimate partner.

## Discussion

Adolescent girls in conflict settings are an especially vulnerable population. Conflict and displacement can exacerbate food insecurity, interrupt medical care, reduce family resources and communal cohesion, and increase exposure to interpersonal violence (Mels *et al*. 2010). Violence prevention and response efforts in conflict settings often focus on violence against adult women rather than girls (Bass *et al*. 2013; Gupta *et al*. 2013). Yet, in our study, nearly one in four girls aged 10–14 reported sexual violence victimization in the last 12 months – a rate similar to the lifetime prevalence of childhood sexual abuse for girls under age 18 in developed contexts (Finkelhor *et al*. 1990), and of sexual violence victimization among women ages 15–49 in the DRC (Peterman *et al*. 2011). These findings demonstrate that girls age 10–14 are particularly vulnerable and that experiences of violence are associated with lowered hope among these girls. As such, their violence exposure combined with low reported future orientation scores indicate a need for interventions to build resilience amongst this population.

Our findings further reveal that attitudes toward IPV have a moderating effect on the relationship between some forms of violence exposure and hope. Specifically, adolescent girls who reported IPV to be acceptable have significantly lower hope scores given exposure to physical violence, unwanted sexual touching, and forced or non-consensual sex, even after adjusting for factors such as age and educational status. These findings indicate that ascription to prescriptive gender norms have negative effects on hope for girls who have experienced physical and sexual violence victimization.

In role congruity theory, violence may be used as punishment for violating gender roles (Diekman & Goodfriend, 2006). Our study indicates that the relationship between descriptive norms (violence experienced) and prescriptive norms (belief in acceptability of violence) has an inverse relationship to hope. In other words, agreement with attitudes accepting of IPV, coupled with physical or sexual violence victimization, indicates a more negative future orientation (including a view of future self). Following role congruity theory, we might hypothesize that girls who experience violence and accept traditional gendered ideas about IPV may internalize beliefs of inferiority and feel that they ‘deserve’ violence. This may in turn contribute to feelings of low self-worth and low hope for the future. Future studies might usefully explore these pathways.

These findings have important implications for violence prevention and response programming. First, community campaigns and targeted programming have tended in recent years to focus on shifting attitudes and norms about violence as a form of primary prevention (Heise, 2011). Our findings suggest that such programming may facilitate tertiary prevention efforts as well, minimizing impact of sexual violence and restoring health and wellness more quickly (Chrisler & Ferguson, 2006). Even when empowerment programs have shown little demonstrated effect on reducing exposure to violence (Heise, 2011), these programs often include activities that challenge traditional gender norms and acceptability of domestic or IPV. Our findings suggest that addressing gender norms may be protective after experiencing some forms of physical and sexual violence in adolescent populations. While primary prevention efforts targeted to girls and influential people in their lives remain critical, potential secondary effects of empowerment programs, such as changing attitudes toward gender norms, may foster resilience in the face of violence and should not be discounted.

Various studies, including the present research, have identified associations between exposure to violence and adverse mental health outcomes. A systematic review of risk and protective factors for children affected by conflict found that ‘[e]xposure to violence is the factor with the strongest evidence base for the risk of subsequent psychological disturbances’ (Reed *et al*. 2012). Given the high prevalence of violence exposure for girls in conflict-affected settings, known associations between violence exposure and adverse mental health outcomes, and evidence of the significant burden of mental disorders amongst children affected by armed conflict (Attanayake *et al*. 2009), improved understanding of factors that may ameliorate or worsen well-being for adolescent girls in these contexts is vital. Conflict-affected settings are replete risk factors for adverse mental health outcomes for girls, making improved understanding of processes and pathways toward mental health outcomes a key research priority (Tol *et al*. 2011). Poor mental health in adolescence remains a public health priority given associations with risky sexual behavior and substance and alcohol use, as well as life-long adverse health outcomes (Patel *et al*. 2007).

A growing body of literature has noted the importance of understanding resilience in the context of adversity experienced by adolescents affected by war (for example, Tol *et al*. 2013). A focus on resilience in situations of armed conflict can shift focus from a ‘deficits’ model – assuming that children will experience mental health problems – toward focusing on the resources children, families and communities engage to address problems.

The findings from the present study also have implications for mental health and psychosocial programming for conflict-affected adolescent girls. Hope itself may be a key component of resilience, and hope has been proposed as one of five empirically supported principles that should be used to guide intervention development to address traumatic experiences, prevent adverse mental health outcomes and promote well-being (Hobfoll *et al*. 2007). Psychosocial interventions – specifically, classroom-based interventions implemented for conflict-affected children in Burundi, Indonesia, Nepal, and Sri Lanka – have shown positive impacts on hope, and demonstrated benefits as a preventative intervention (for example, Jordans *et al*. 2010; Tol *et al*. 2014). A systematic review of the impact of mental health and psychosocial interventions on conflict-affected children has similarly identified effective approaches targeting individual children, yet indicates a need to further focus on strengthening community and family supports (Jordans *et al*. 2016). Indeed, while mental health and psychosocial services in humanitarian settings often focus on individual clinical outcomes, evidence points to the need to address risk factors that operate at family and community-levels (Miller & Jordans, 2016). Given the relationships between violence exposure, prescriptive gender norms and hope identified in our study, findings support the potential for development of combined violence prevention and holistic psychosocial interventions to improve adolescent girls’ resilience in situations of extreme adversity.

Limitations of this study include using a single indicator for physical and emotional violence. The cross-sectional nature of this study limits ability to confirm temporal associations. It is possible that the direction of association between the variables examined may not be as hypothesized; however, other studies have found that attitudes toward IPV precede adolescent violence exposure (Lichter & McCloskey, 2004). Also lending support to our hypothesis, this study's violence indicators asked about past-year exposure, while indicators for hope measured feelings at the time of the study. While the authors attempted to account for community-level factors that may influence attitudes toward IPV through inclusion of district-level variables, there may have been other individual-level factors that influenced attitudes toward IPV and hope that were not measured. Lastly, the study sample may have been subject to selection bias, since selection criteria included enrollment in a life skills program, and may have led to exclusion of girls with more limited access or support to participate in the program.

## Conclusion

Promotion of equitable gender norms in conflict-affected settings has been found to be a promising practice to reduce violence against women (Gupta *et al*. 2013). Future research might usefully explore whether these programs have secondary effects on reducing stigma and improving hope among adolescent girls who experience sexual violence. Continued financial, programming, and research investments must be made to promote the empowerment, resilience, and safety of adolescent girls in eastern DRC and other fragile contexts. Addressing accepting attitudes toward IPV may be one such mechanism to improve the prevention and response to violence against adolescent girls in conflict-affected settings.
